# Lateral Thoracodorsal Flap Revisited: An Underappreciated Workhorse

**DOI:** 10.29252/wjps.9.2.212

**Published:** 2020-05

**Authors:** Pavneet Kohli, Prasanth Penumadu, Kadambari Dharanipragada, MT Friji

**Affiliations:** 1 Department of Surgical Oncology, Jawaharlal Institute of Postgraduate Medical Education and Research (JIPMER), Pondicherry, India;; 2 Department of General Surgery, Jawaharlal Institute of Postgraduate Medical Education and Research (JIPMER), Pondicherry, India;; 3 Department of Plastic Surgery, Jawaharlal Institute of Postgraduate Medical Education and Research (JIPMER), Pondicherry, India

**Keywords:** Breast, Conservative surgery, Oncoplastic surgery, Volume replacement, Thoracodorsal flap

## Abstract

**BACKGROUND:**

Although, the lateral thoracodorsal flap is a well described technique, its utility seems to be lost in the ever evolving world of oncoplastic breast surgery. This study reviews the technique, its indications and limitations and the advantage of this technique.

**METHODS:**

Between January 2016 and January 2018, data from 7 consecutive patients who underwent partial breast mastectomy with lateral thoracodorsal flap were enrolled. A wedge shaped flap was designed using the pinch test using the index finger and the thumb in small defects, while larger defects required a convex shaped incision with curved superior and inferior borders. Incision was made along the marked margins of the proposed flap and deepened to the underlying serratus anterior and latissimus dorsii muscle. The flap was transposed in the defect and the symmetry of mound between the two breasts confirmed in sitting and supine position.

**RESULTS:**

All patients were satisfied by cosmetic outcomes on visual analog scale (VAS). Cosmetic results based on Harvard scale showed good to excellent scores. Evaluation by Breast Cancer Conservation Treatment (BCCT) core software illustrated good to excellent cosmetic outcomes. There was no delayed wound healing, marginal skin ornecrosis and no evidence of any fat necrosis in the follow up period.

**CONCLUSION:**

The versatility of latissimus dorsii flap, good aesthetic and functional results and its simple execution made it an important option in the armamentarium of the oncoplastic breast surgeon. Also, morbidity of the donor site was minimized without sacrificing muscles or nerves.

## INTRODUCTION

Breast conservative surgery (BCS) along with post-operative radio-therapy (PORT) has become the standard of care for early breast cancers (EBC), resulting in similar overall survival and better quality of life scores.^1^^-^^4^ Oncoplasty breast surgery (OPS) is a new addition to the ever evolving armamentarium of breast surgeons. The technique of current day BCSs is a paradigm shift from the National Surgical Adjuvant Breast and Bowel Project (NSABP) guidelines of 1987.^5^^,^^6^ OPS today include diverse techniques both volume displacement and volume replacement, abiding with current oncological principles and simultaneously helping in achieving good to excellent cosmetic results.^7^

Despite the rapid growth of this field and the vast number of techniques described for tumors in specific quadrants. It is accepted that 10-30% of patients undergoing BCS are unsatisfied with the cosmetic outcomes.^8^^-^^10^ The main reasons are related to tumor excision, which can lead to asymmetry, a visible scar, volume changes and nipple retraction.^11^^,^^12^ New techniques have been described, so as to cater to the increased demands for reduced scars. This has led to development of several techniques with minimal incisions and periareolar incisions.^13^^,^^14^

However, most of these are suited for a limited group of cases. Suboptimal aesthetic outcomes are also related to poor adjustment and inferior quality of life in breast cancer survivors.^15^ While tissue implants and free flaps are now the order of the day in the West, reconstruction using autologous local flaps [like the latissimus dorsii myocuatenous flap, the Transverse rectus abdominis myocutaneous (TRAM) flap] still form the major form of reconstruction in resource limited countries like India due to both cost and social issues.

The lateral thoracodorsal flap was first described in the 1980s by Holmstrom and Lossing.^16^ It is an implant-based technique using local flaps for delayed post-mastectomy breast reconstruction, and is a versatile flap which is often underappreciated and under used in the setting of immediate breast reconstruction after partial mastectomy defects. Although the lateral thoracodorsal flap is a well described technique, its utility seems to be lost in the ever evolving world of OBS. This case series reviews the technique, its indications and limitations and aims to emphasize the advantage of this technique and its usefulness as an addition to the armamentarium of a breast surgeon.

## MATERIALS AND METHODS

Between January 2016 and January 2018, data from 7 consecutive patients who underwent partial breast mastectomy with lateral thoracodorsal flap in Jawaharlal Institute of Postgraduate Medical Education and Research (JIPMER), a tertiary hospital in South India was evaluated retrospectively. The study was approved in the institution ethics committee. All patients were first seen in the preoperative period by a multidisciplinary tumor board. Breast volume, presence of ptosis, and tumor size/location were evaluated by a plastic surgeon, who indicated the immediate reconstruction with the appropriate technique for each case. A written consent was provided from each patient.

All surgeries were performed by a single plastic surgeon and under general anesthesia. Axillary dissection was performed in all patients. From the patients’ medical and surgical records, information such as comorbidity stages, age, and body mass index, history of smoking or radiation treatment, and complications were obtained and assessed. Oncological outcomes like margin status, number of nodes dissected were also studied. A satisfaction survey was conducted among patients and the surgeon, both immediately and after 6 months.

Regarding surgical technique, pre-op planning was started in the out-patient department at time of first presentation. Clinical examination with aid of calipers and pre-op photographs were used as per standard practice. Skin changes like Peau d’orange, puckering, dimpling, Nipple-Areolar Complex (NAC) involvement were a contraindication for surgery. Only lateral breast tumors were considered for this form of reconstruction. A bilateral mammography and an informed consent were essential before surgery.

Marking was done in a well-lit pre-op suite in sitting and lateral decubitus position. The inferior mammary fold, the central meridian of the breast and the anterior axillary line was identified and marked in the sitting position. The posterior axillary line and the boundaries of latissimus dorsi (LD) muscle were marked as well in case defect was bigger than anticipated and needed a LD flap for reconstruction. Though the flap has been described in the lateral and semi lateral decubitus position, we performed all reconstruction in a supine position with a sand bag placed underneath the ipsilateral shoulder, thus saving operating time. A formal aesthetic evaluation of the breast was done by using a visual analog scale (VAS) score, Harvard scale^17^ and using the Breast Cancer Conservation Treatment (BCCT) core software.^18^

**Fig. 1:**
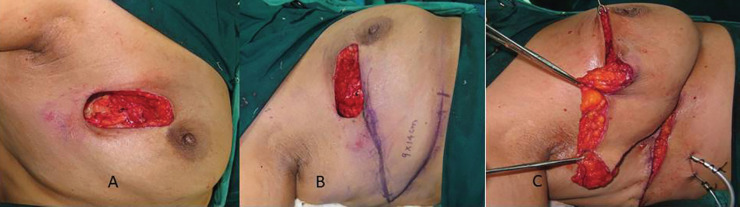
**A. **The defect after the tumor is resected as per oncological principles. Skin is not involved.** B. **A wedge shaped lateral thoracodorsal flap is marked with the infra-mammary fold forming its axis.** C. **The flap is advanced, the skin de-epithelialized to provide bulk to the defect

The infra-mammary fold and lateral and dorsal extension along it formed the axis of the flap, while the anterior axillary line formed the base, taking it into consideration that the final scar would be hidden beneath the brassiere (Figure 1). A wedge shaped flap was designed using the pinch test using the index finger and the thumb in small defects, while larger defects required a convex shaped incision with curved superior and inferior borders to fill in the defect. The base of the flap can vary depending on the defect, the fat and subcutaneous tissue available and can reach up to 5-10 cm. The length of the pedicle can vary from 10-15 cm. 

We did not use Doppler on regular basis. The surgeon stands on the ipsilateral side and performs the excision taking into consideration the oncological principles. Axilla dissection is performed through the same incision in small breasts or through a different incision in larger breasts. Incision is made along the marked margins of the proposed flap and deepened to the underlying serratus anterior and latissimus dorsii muscle, elevating the flap subfascially. The flap is based on the lateral intercostal perforator artery and hence skin and subcutaneous tissue are dissected away from the muscles in a lateral to medial manner. 

**Fig. 2:**
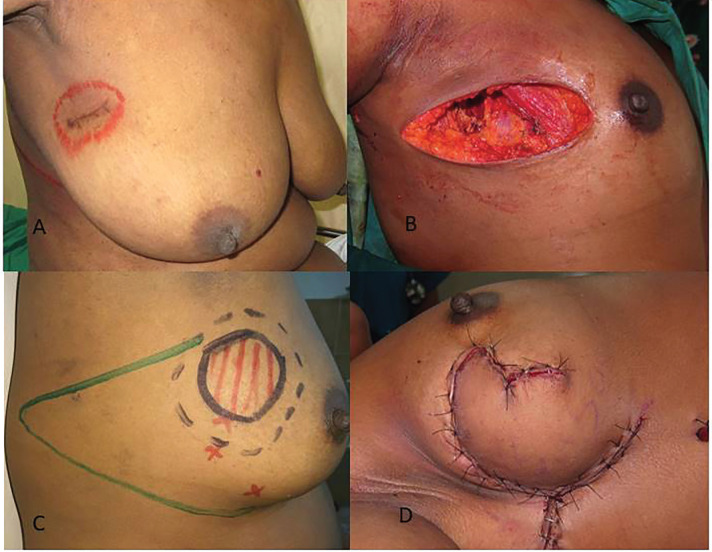
**A. **Scar of excision biopsy for a right breast lump at a primary center with margin status unknown. **B. **Defect after excision of tumor along with overlying skin.** C. **Pre-operative marking of The LTDF flap.** D. **Final result after the LTDF flap has been transposed to provide skin as well as soft tissue coverage

**Fig. 3:**
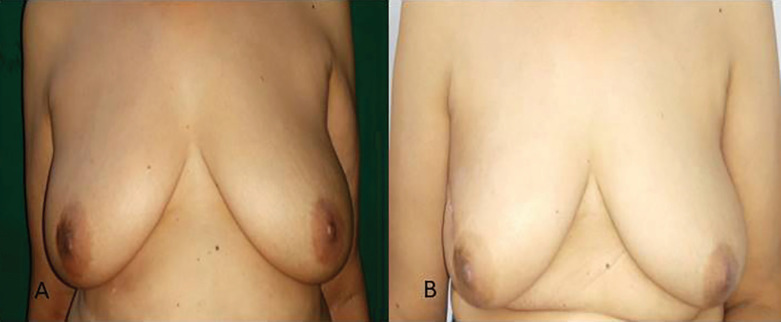
Cosmetic outcome after lateral thoracodorsal flap.** A. **Pre-operative picture with tumor in upper outer quadrant of left breast. **B. **Post-operative results after 6 months

Extra care is taken not to breach the fascia at the junction of latissimus dorsi and serratus anterior muscle. The flap was transposed in the defect and the symmetry of mound between the two breasts confirmed in sitting and supine position (Figure 2). Necessary de-epithelialization and tissue rearrangement are done to achieve good results and a drain placed. The flap can be transferred by an advancement or transposition technique to achieve a good cosmetic result (Figure 3). 

## RESULTS

Between July, 2016 and January 2018, seven females were selected to undergo reconstruction by lateral thoracodorsal flap in the Department of Surgical Oncology, Surgery and Plastic Surgery, JIPMER, Pondicherry, a tertiary health care center in South India. Two patients with breast cancer underwent neoadjuvant chemotherapy to make them eligible for BCS. The mean age of patients was 58.85±15.30 years (range: 30-76 years). The tumors were located in the upper outer quadrant in 4 and in the lower outer quadrant in 3 patients. 

The left breast was affected in 2 patients and the right breast in 5 patients. The mean tumor size of patients with breast cancer was 2.68±1.21 cm (range: 1.3-5.0 cm). There were 3 patients with pT1, 3 patients with pT2, and 1 patient with pT3 tumors. The median duration of follow up was 528 days. One patient had a distant recurrence in this period of follow up. Pre-operative results with tumor in upper outer quadrant of left breast and post-operative results after 6 months using lateral thoracodorsal flap and the cosmetic outcome were shown in Figure 3.

**Fig. 4:**
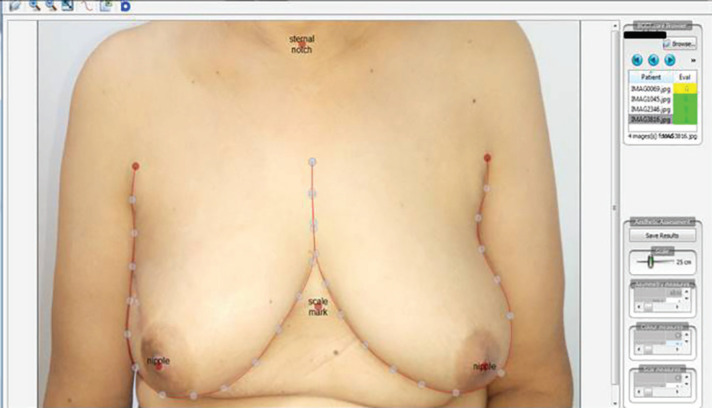
Use of Breast Cancer Conservation Treatment (BCCT) core software^18^ to assess cosmetic outcome

Figure 2 demonstrates scar of excision biopsy for a right breast lump at a primary center with the unknown margin status. Also, the defect after excision of tumor along with overlying skin and pre-operative marking of The latissimus dorsi flap (LTDF) and the final result after the LTDF transposed to provide skin as well as soft tissue coverage were illustrated in Figure 2. All patients were satisfied by the cosmetic outcomes on VAS score. The cosmetic result based on Harvard Scale showed good to excellent score in all patients. Evaluation by BCCT core software showed cosmetic outcome to be good to excellent score (Figure 4). None of the patients had a positive margin. Surgical site infections were the commonest complication and were seen in one patient. There was no delayed wound healing, marginal skin or NAC necrosis. There was no evidence of any fat necrosis in the follow up period (Table 1).

**Table 1:** Demographic data of patients who underwent Lateral thoracodorsal flap

**No.**	**Initials**	**Age**	**Size**	**Margins**	**Quadrant**	**Side**	**T stage**	**Hormonal status**	**NACT received**	**Complications**	**VAS score**	**Harvard Score**	**BCCT Core Score**
1	SD	76	2.5 cm	Negative	Upper Outer	Left	T2	Positive	No	None	9	Excellent	Excellent
2	SR	74	3.2 cm	Negative	Upper Outer	Right	T2	Positive	Yes	None	9	Excellent	Excellent
3	RD	63	1.8	Negative	Lower Outer	Right	T1	Positive	No	None	8	Good	Good
4	PR	56	3	Negative	Upper Outer	Right	T2	Positive	No	SSI and Wound dehiscence	8	Good	Good
5	KP	59	2	Negative	Lower Outer	Right	T1	Negative	No	None	9	Excellent	Excellent
6	MR	30	5	Negative	Lower Outer	Right	T3	Negative	No	None	8	Good	Good
7	CA	54	1.3	Negative	Upper Outer	Right	T1	Negative	Yes	None	9	Excellent	Excellent

## DISCUSSION

The field of oncoplasty is an ever evolving one, with various techniques, described, accepted and practiced depending on the ingenuity of the surgeon. We believe that this technique though well described is often overlooked and forgotten in lieu of the more morbid LD or mini LD flaps.^19^ It is a simple yet an important addition to the versatility of a breast surgeon when dealing with breast malignancies. The technique works well for outer quadrant (upper and lower), central and retro areolar tumors not involving the NAC.

The essence of the procedure is that it allows excision of tumor without compromising on the oncological aspects. This technique provides better cosmesis. The axis of the flap is set along the line of the inframammary fold, extending toward the lateral and posterior sides, and the base of the flap is designed to lie on the anterior axillary line, so that the postoperative scar could be hidden under the brassiere line**. **Overall, there is a high degree of cosmetic satisfaction amongst patients, which is suitable in patients demanding minimal visible scarring in breast surgery.

Axillary clearance can be done through the same incision in the same position and same setting. Learning curve of the procedure is not steep. There is no increase in operating time and no great effect of co morbidities on choice of surgery and there is no need to change position during surgery. The technique decreases the need for the classical LD flap reconstruction and morbidity associated with it. It also allows easy switch to other volume replacement techniques like LD flap if the need arises. The versatility of LTDF and its simple execution made it an important option in the armamentarium of the oncoplastic breast surgeon. The LTDF procedure has the advantage of good aesthetic and functional results being similar in texture and color to the native breast; in addition, morbidity of the donor site is minimized without sacrificing muscles or nerves. 

## CONFLICT OF INTEREST

The authors declare no conflict of interest.
